# Factors influencing uptake of telemental health via videoconferencing at high and low adoption sites within the Department of Veterans Affairs during COVID-19: a qualitative study

**DOI:** 10.1186/s43058-022-00318-x

**Published:** 2022-06-20

**Authors:** Samantha L. Connolly, Jennifer L. Sullivan, Jan A. Lindsay, Stephanie L. Shimada, Leonie Heyworth, Kendra R. Weaver, Christopher J. Miller

**Affiliations:** 1 Center for Healthcare Organization and Implementation Research (CHOIR), VA Boston Healthcare System, Boston, MA USA; 2 Department of Psychiatry, Harvard Medical School, Boston, MA USA; 3 Center of Innovation in Long Term Services and Supports, VA Providence Healthcare System, Providence, RI USA; 4 Brown University School of Public Health, Providence, RI USA; 5 Michael E. DeBakey VA Medical Center, HSR&D Center for Innovations in Quality, Effectiveness and Safety, Houston, TX USA; 6 Baylor College of Medicine, Houston, TX USA; 7 South Central Mental Illness Research, Education and Clinical Center, Houston, TX USA; 8 Department of Health Law, Policy and Management, Boston University School of Public Health, Boston, MA USA; 9 Center for Healthcare Organization and Implementation Research (CHOIR), VA Bedford Healthcare System, Bedford, MA USA; 10 Division of Health Informatics and Implementation Science, Department of Population and Quantitative Health Sciences, University of Massachusetts Medical School, Worcester, MA USA; 11 Veterans Health Administration Office of Connected Care/Telehealth, Washington, D.C, USA; 12 Department of Health Sciences, University of California San Diego, San Diego, CA USA; 13 Clinical Operations, Veterans Health Administration Office of Mental Health and Suicide Prevention, Washington, D.C, USA

**Keywords:** Telehealth, Mental health, Telemental health, Telepsychiatry, CFIR, Qualitative

## Abstract

**Background:**

The coronavirus disease 2019 (COVID-19) pandemic dramatically increased the use of telemental health via videoconferencing (TMH-V). While TMH-V has been found to be effective and satisfactory to both patients and providers, little is known regarding factors that influence site-level uptake. We examined facilitators and barriers to TMH-V uptake at higher and lower adoption sites within the US Department of Veterans Affairs (VA).

**Methods:**

We conducted twenty-four semi-structured qualitative interviews at four northeastern VA medical centers (two with higher TMH-V adoption and two with lower adoption). Six interviews were conducted per site (one member of mental health leadership, one facility telehealth coordinator/technician, and four mental health providers per site). We performed directed content analysis, guided by the Consolidated Framework for Implementation Research (CFIR), followed by a matrix rating process to rank the degree of influence of each of the 19 included CFIR constructs at the four sites. Positive overall influences, negative overall influences, and differentiators were then identified based on patterns in ratings across sites.

**Results:**

Five CFIR constructs had positive overall influences across sites: Relative advantage, Patient needs and resources, Relative priority, Knowledge and beliefs, and Self-efficacy. Complexity had a negative overall influence across sites. Four constructs significantly differentiated between higher and lower adoption sites with regards to TMH-V use: Quality, Compatibility, Leadership engagement, and Champions.

**Conclusions:**

Several positive overall influences on TMH-V uptake were identified across sites; respondents acknowledged multiple advantages of TMH-V (e.g., convenience), and providers’ attitudes towards TMH-V improved as they gained experience. In contrast, complexity was a negative overall influence; TMH-V platforms and processes must be simple and user friendly to promote use. The emergence of Quality, Leadership engagement, and Champions as differentiators speaks to the importance of educating frontline staff and leadership at lower adoption sites about the evidence base demonstrating that TMH-V is high-quality care. Compatibility also emerged as a differentiator; if TMH-V is not easily integrated into provider workflows, uptake will falter. Future work should draw from these findings to develop implementation strategies aiming to increase TMH-V uptake at lower adoption sites, thereby increasing access to high-quality mental health care.

**Supplementary Information:**

The online version contains supplementary material available at 10.1186/s43058-022-00318-x.

Contributions to the literature

- While there was a rapid increase in telemental health via videoconferencing (TMH-V) during COVID-19, little is known about site-level variability in uptake.
- Guided by the Consolidated Framework for Implementation Research (CFIR), we found multiple factors that served as facilitators and barriers to TMH-V uptake. Findings point to the importance of education regarding the high quality of TMH-V care, the need for hands-on support from leadership, and the seamless integration of TMH-V into clinical workflows to ensure implementation success.
- Ideally, future work can draw from these findings to develop implementation strategies to increase TMH-V uptake at lower adoption sites.

## Background

Telemental health via videoconferencing (TMH-V) allows care to be delivered in real-time to a patient’s home or other personal location and can increase access to vital mental health (MH) services. The US Department of Veterans Affairs (VA) has long been a champion of telehealth given its mission to provide care to Veterans living in all regions of the US; indeed, VA was the first healthcare system to appoint a chief telehealth officer over twenty years ago [1]. Following the advent of its telehealth-to-home platform, VA Video Connect, in 2017, VA set national goals aiming to increase the number of MH providers who had completed at least one TMH-V appointment by the end of 2019 [2]. Although usage rates did increase due to this initiative, it did not lead to a substantial shift towards remote care, due to factors such as patient and provider reticence as well as workflow challenges (e.g., difficulties adjusting to new scheduling processes [3].

The coronavirus disease 2019 (COVID-19) pandemic led to a rapid and unprecedented increase in TMH-V, as the need to provide MH services while protecting patients and providers from infection overcame many of the abovementioned barriers to adoption. Indeed, VA demonstrated 556% growth in TMH-V visits in the early months of COVID-19 [1–3]. A growing body of research has begun to characterize this dramatic transition to TMH-V care from the provider perspective, including the need to quickly adapt to new and often complex technologies, the importance of being flexible and creative in converting care delivery to virtual formats, and the finding that TMH-V was often more effective and satisfactory than providers had initially expected [4–8]. Findings are also emerging that patients are largely satisfied with TMH-V, in that they view it as high quality and often more convenient than in-person care [9–11].

However, little is known regarding site-level variability in TMH-V uptake during COVID-19. While increased TMH-V use was nearly universal across VA, rates of uptake varied across facilities, particularly given that a large proportion of remote care was also being delivered via audio-only phone visits [2]. Phone visits have significantly fewer barriers to use, given that they do not require patients to have video-enabled devices or internet connectivity to engage in care [12]. In addition, providers and leadership may feel that in-person care is higher quality than remote care, particularly for patients with more severe symptoms, which may lead to lower levels of TMH-V use at a site [13, 14]. The degree of infrastructure in place to support TMH-V is likely also an important contributor to uptake (e.g., availability of technical support staff, streamlined scheduling processes) [15].

A national quantitative study of site-level predictors of TMH-V use during COVID-19 within VA found that sites with poorer broadband coverage and less telehealth experience prior to the pandemic demonstrated lower rates of use [16]. However, to our knowledge, there has yet to be a qualitative study of key stakeholders’ perceptions of facilitators and barriers to TMH-V uptake across sites with higher and lower adoption rates. The current study sought to fill this gap, guided by the Consolidated Framework for Implementation Research (CFIR) [17], which includes a selection of domains and constructs that can influence uptake of innovations into practice, informed by a rigorous review of the implementation science research literature. Nuanced identification of facilitators and barriers to TMH-V use at both higher and lower adoption sites will be critical in informing sustained use of TMH-V well beyond the COVID-19 pandemic.

## Methods

### Facility selection

Four sites were selected from eight total VA medical centers in a northeastern region of the US. Sites were chosen based on the percentage of mental health (MH) providers who had completed at least one TMH-V visit as of June 2020, a metric that is tracked for operational purposes within the national Veterans Health Administration Support Service Center (VSSC) database. Given the onset of COVID-19 and restrictions on in-person MH care, rates of TMH-V experience across all medical centers were generally high; we chose the two sites with the highest adoption rates (Site 1: 97.7%, Site 2: 94.5%) and the two sites with the lowest adoption (Site 3: 83.6%, Site 4: 84.4%)^1^.

### Study participants

Twenty-four employees participated in qualitative interviews between March and October 2020: sixty employees were approached (40% participation rate). Six interviews were completed per site, including one telehealth coordinator or technician, one member of MH leadership (MH lead, psychology lead, chief of psychiatry, MH service chief), and four MH providers (six total psychiatrists, five psychologists, four social workers, and one physician assistant). Telehealth coordinators and technicians were identified via a centralized list supplied by the regional VA Office of Connected Care. Providers were identified via the VSSC database, which lists providers’ names, discipline, and number of completed TMH-V visits since 2017. This allowed for recruitment of providers with both high and low levels of TMH-V use (range: 9—453 TMH-V visits)^2^. MH leadership were identified via medical center websites and provider reports of current leadership structure at their sites.

### Measures

A semi-structured interview guide was developed based on the CFIR, with questions grouped within the five CFIR domains (Intervention characteristics, Outer setting, Inner setting, Characteristics of individuals, Process). While informed by these CFIR domains, the interview guide also allowed for more general, open-ended discussion of stakeholders’ impressions of and attitudes toward TMH-V use at their site including barriers and facilitators that may have impacted TMH-V adoption. The semi-structured format of the guide allowed for participants to expand upon any specific facilitators and barriers that they found to be important**.** SLC developed the initial draft of the interview guide and edited it based on feedback from JLS and CJM (see Additional file 1 for full interview guide).

### Procedure

Stakeholders participated in hour-long semi-structured qualitative interviews conducted via telephone by SLC. Interviews were audio-recorded and professionally transcribed. This study was deemed Institutional Review Board exempt by the VA Boston Research and Development Committee, and a waiver of informed consent was obtained. Participants provided verbal informed consent prior to interview initiation, and participation was voluntary.

### Data coding and analysis

We conducted directed content analysis [18] with the CFIR serving as the coding framework. SLC developed a codebook that included 19 CFIR constructs across the five CFIR domains; constructs were chosen from the 39 total CFIR constructs based on applicability to current research aims and the preexisting research base on telehealth implementation [19–22]. SLC proposed an initial set of potential CFIR constructs to include, and the finalized list was determined based on all co-authors’ input, given their range of expertise in implementation science and TMH-V use. SLC coded the 24 interviews using NVivo 12 software [23], during which she modified code definitions and added emergent codes, resulting in 28 total codes (see Additional file 2 for list of codes). CJM and JLS then reviewed and provided feedback on this codebook, after which the group came to consensus on the finalized codebook. CJM and JLS double-coded 12 transcripts each; any discrepancies or disagreements in coding decisions were discussed by the coders to achieve consensus, after which all coding was finalized.

SLC reviewed all relevant coded segments for each CFIR construct on a per-site basis, after which she developed a summary of key findings and assigned a rating to indicate the degree of influence the construct had on TMH-V implementation at each site. Damschroder et al.’s [24] rating system was used (−2, −1, 0, 1, 2), with negative numbers indicating “negative influence in the organization, an impeding influence in work processes, and/or an impeding influence in implementation efforts” and positive numbers indicating “positive influence in the organization, a facilitating influence in work processes, and/or a facilitating influence in implementation efforts.” Ratings were coded as missing if there were insufficient data available at a given site (less than two respondents per code; see Additional file 3 for full rating criteria). This resulted in a matrix of scores for the 19 CFIR constructs across all four sites (the additional emergent codes were not scored as they were not part of CFIR). The matrix included all quotes that informed the rating, as derived from the above-described double coding process, as well as a written rationale summarizing the rating made by SLC. This matrix was then reviewed by JLS and CJM. A consensus discussion was then conducted; in cases where there were any discrepancies regarding the rating, SLC would refer back to supporting evidence and the transcripts. This new information was then reviewed by the full team until consensus on all ratings was reached.

SLC then assessed for patterns in ratings that differentiated between low and high adoption sites. A construct was determined to be a *positive overall influence* if at least three of the four sites had a positive rating and no sites had a negative rating. Conversely, a construct was a *negative overall influence* if at least three of the four sites had a negative rating and no sites had a positive rating. A construct was determined to be a *differentiator* if a consistent pattern emerged at the majority of sites (i.e., three out of four sites) such that higher-adoption sites had higher scores and lower-adoption sites had lower scores, with no contradictory ratings. For instance, a construct would be a differentiator if both of the lower-adoption sites had negative scores, at least one of the higher-adoption sites had a positive score, and none of the higher-adoption sites had a negative score. CJM and JLS reviewed this determination of positive overall influences, negative overall influences, and differentiators, and consensus was reached across the qualitative team.

We took several steps to strengthen the credibility of analyses, in accordance with the Standards for Reporting Qualitative Research (SRQR) [25]. SLC, JLS, and CJM have substantial experience within the fields of mental health, implementation science, and qualitative research within VA; SLC and CJM are clinical psychologists, and JLS is an implementation scientist. This prolonged engagement allowed for improved data interpretation abilities. SLC has intermediate training in qualitative methodologies, and JLS and CJM have additional advanced training in qualitative research and have extensive experience leading and participating in qualitative studies.

All research procedures and analytic decisions were thoroughly documented and discussed during the development of this work. In addition, SLC, JLS, and CJM met to achieve consensus with regards to the interview guide, coding, and matrix ratings to help reduce bias. (See Additional file 4 for full SRQR checklist).

## Results

Of the 19 included CFIR constructs, five had positive overall influences across sites: Relative advantage, Patient needs and resources, Relative priority, Knowledge and beliefs, and Self-efficacy. Complexity had a negative overall influence across sites. Four constructs significantly differentiated between higher and lower adoption sites with regards to TMH-V use: Quality, Compatibility, Leadership engagement, and Champions. The remaining nine constructs either showed inconsistent rating patterns across sites or had missing data for at least one site (see Table 1). Positive overall influences, negative overall influences, and differentiators are discussed below along with representative quotes.

**Table 1:** Site-level CFIR rating matrix

CFIR domain/construct	Site rating				Differentiator, positive overall, negative overall
	Higher adoption sites		Lower adoption sites		
	Site 1	Site 2	Site 3	Site 4	
**I. Intervention characteristics**					
Complexity	− 2	− 2	− 2	− 2	Negative overall
Quality	2	1	0	0	Differentiator
Relative advantage	1	1	2	0	Positive overall
**II. Outer setting**					
External policies and incentives	1	0	0	1	
Patient needs and resources	1	1	1	0	Positive overall
**III. Inner setting**					
Access to knowledge and info	1	0	1	− 2	
Available resources	0	1	1	− 1	
Compatibility	1	0	− 1	− 1	Differentiator
Culture	2	0	Missing	Missing	
Goals and feedback	Missing	0	1	1	
Implementation climate	1	1	Missing	Missing	
Incentives and rewards	0	Missing	0	Missing	
Leadership engagement	2	2	0	− 1	Differentiator
Networks and communication	2	Missing	Missing	− 1	
Relative priority	1	1	0	1	Positive overall
**IV. Characteristics of individuals**					
Knowledge and beliefs	1	1	1	0	Positive overall
Self-efficacy	1	0	1	1	Positive overall
**V. Process**					
Champions	2	1	0	0	Differentiator
Planning	1	Missing	0	Missing	

*Note*. See Additional file 3 for Damschroder et al.’s numeric rating criteria. A construct was determined to be positive overall if at least three of the four sites had a positive rating and no sites had a negative rating. Conversely, negative overall indicates that at least three of the four sites had a negative rating and no sites had a positive rating. A construct was determined to be a differentiator if a consistent pattern emerged in at least three of the four sites, with no contradictory ratings (e.g., both of the lower-adoption sites had negative scores, at least one of the higher-adoption sites had a positive score, and none of the higher-adoption sites had a negative score)

*CFIR* Consolidated Framework for Implementation Research

### Positive overall influences

#### Relative advantage

Sites were generally in agreement regarding the relative advantages conferred by TMH-V, specifically with regards to increased access and convenience for patients (e.g., not having to drive to visits).

[Some of my patients are] two hours one way so a four-hour drive [roundtrip]…there’s that balance of, if I want to do therapy with someone, I need to not be adding so much stress to their life that they’re already overwhelmed when they show up at my office… [Site 2, social worker]

Respondents described that TMH-V also allows for more flexibility for patients with work and childcare responsibilities, and may serve as a steppingstone into therapy for patients with severe anxiety or stigma surrounding in-person care.

Women veterans don’t always feel safe coming to VA. Some of them are military sexual trauma survivors, some of them [have] severe PTSD (posttraumatic stress disorder). But also…new moms or [those who] have school age kids…they're running a household and so being able to [attend sessions] right from their home saves them the stress of having to find a sitter… [Site 1, facility telehealth coordinator]

Some noted the advantages of video over phone in terms of being able to see the patient and their home environment, as well as meet family members in some cases. Others described significant space issues at their facilities that made TMH-V particularly advantageous over in-person care. Infection prevention during COVID-19 was also noted as a benefit of TMH-V care.

#### Patient needs and resources

Respondents across all sites noted that many patients preferred TMH-V, for the various reasons outlined in the relative advantage section above. Respondents were also aware of significant barriers to TMH-V use for certain patients, including those without access to video-enabled devices or internet connectivity, or those with lower technological literacy. Older, rural, and lower-income veterans were often noted as falling into these categories, although several respondents were quick to point out exceptions to these generalizations (e.g., veterans in their nineties who had embraced TMH-V sessions).

Respondents at three of the four sites were aware of a VA program to help overcome structural barriers to access by sending internet-enabled tablets to veterans without devices. Sites varied in the availability of training resources for patients; telehealth technicians at some sites had more time to assist patients with test calls and troubleshooting as opposed to others:

I’d say the majority [of patients] like [TMH-V]. Some have needed more help with the technology, but…if I can’t train them or have the resident train the Veteran to do the video, the [telehealth technicians] have. So…even those that [initially] said, ‘No, I’ll just do it by phone,’ I’m pleasantly surprised that they’re…now agreeing to do it by video. [Site 3, psychiatrist]

#### Relative priority

Sites generally agreed that increasing TMH-V use was a priority at their site, particularly over the use of phone care. For some sites, this was described as a major focus:

[Increasing TMH-V use is] a pretty high priority, especially now with all the COVID stuff…I try to frame it as, this is really the future. So it’s not like people can just ride it out until it’s old news. That's how we and the VA are going to stay relevant. Just kind of putting to people that it's not like a choice unless you’re planning to retire over the next year. [Site 1, MH lead]

Some referenced national initiatives to increase TMH-V use which were in existence prior to COVID-19 and factor into annual site-level performance reviews; this initiative in part increased prioritization of TMH-V use. However, there was also conflicting messaging regarding a push to return to in-person care at certain sites; while these sites agreed that TMH-V use should be prioritized over phone care, they were less certain regarding how much it should be prioritized over in-person visits.

#### Knowledge and beliefs

This CFIR construct refers to individuals’ “attitudes toward and value placed on the intervention.” Many respondents described being initially skeptical regarding whether TMH-V would be effective and whether they and their patients would like using it; however, almost all of these respondents described ultimately being pleasantly surprised by how well TMH-V worked:

I’m more cautious. I like technology but I’m not an expert. Initially…I did not think [TMH-V] could work as well. But [now] having done it…I believe the opposite. [Site 2, social worker]

COVID-19 essentially forced many providers to gain substantial experience with TMH-V due to in-person visit restrictions; this rapid increase in experience appeared to have substantial effects on providers’ beliefs regarding the quality of TMH-V.

#### Self-efficacy

Several staff noted an initial element of provider fear and discomfort in navigating TMH-V technology with patients. However, they described this fear subsiding after gaining experience:

A lot of [providers] are afraid of technology and I think they were afraid it was going to be a lot more trouble than what it’s worth…there is no fear element anymore with the ones that are now doing [TMH-V]… [once they] get past the first couple [sessions] then they’re like pros and they love it. [Site 3, telehealth technician]

Some stakeholders noted their older age as a barrier to technology use, but again the theme emerged that self-efficacy increased with experience:

It was a steep learning curve, and you’re kind of on your own. And I'm almost 65, I’m not a real computer whiz. I’m not an idiot, it’s just I’m not computer savvy…[but TMH-V has] become easier for me. I still have difficulties… [but] I’ve gotten better at it. [Site 4, psychologist]

One provider attributed a large part of their increased self-efficacy to COVID-19, in that they used telehealth with a much greater variety of patients than they would have otherwise:

I think that the more practice you get, the better you get. Like anything. The thing that stands out the most, now that I’m doing [TMH-V] more, I’ve had to take every case on [via video]…regardless of what the presenting problem was…I had to just do it. Whereas, before, when I was doing [TMH-V]…I’d pick and choose and say, well, this person’s higher risk…I don’t want to do that….and I can’t do that anymore. That’s the big difference, is just having the practice…[becoming] more comfortable… having to be okay with accepting risk and…with that greater ambiguity. [Site 1, psychologist]

### Negative overall influence

#### Complexity

Respondents across all sites described the complexity of TMH-V as a major barrier to implementation. They described experiencing complicated scheduling processes, in part influenced by a change in scheduling platform early in the pandemic, as well as difficulties on both the provider and patient side in locating the link to the TMH-V visit; initially links could only be sent via email, although there is now an option to send patient links via text message. Some respondents noted that not all patients had an email address or knew how to set one up, which posed an additional barrier to use.

Multiple respondents noted struggling to help patients troubleshoot technology while at a distance, without being able to see their device in-person and understand the problem:

Sometimes it’s hard for me to help somebody go step-by-step when they are on their phone or…their iPad, I don’t know how to tell them how to turn on their audio if they are having trouble with that. So, I do think the system itself isn’t that user friendly, particularly for folks who are not technologically minded. [Site 2, psychology lead]

Many respondents noted a need for increased support from telehealth technicians to help less tech-savvy patients become comfortable with TMH-V. One respondent noted that without that support, providers may be more likely to convert video visits to phone calls, which are considerably less complex:

We want our practitioners and our veterans to use [TMH-V], but technology is a challenge for them…and we don’t have a solid process of anyone making a test call with them….so I think people just automatically flip to the phone especially since we give them that option…and all is right with the world. [Site 4, facility telehealth coordinator]

### Differentiators

The four constructs described below differentiated between higher and lower adoption sites with regards to TMH-V use.

#### Quality

This CFIR construct is specific to how individuals judge the quality of TMH-V, particularly in comparison to in-person or phone care. Respondents at higher adoption sites more frequently described the quality of care provided via TMH-V as largely equivalent to in-person care, and as higher quality than audio-only phone care:

When I first started using [TMH-V]…I really felt it was probably going to be inferior to a face-to-face visit as far as… body language and developing a connection. But, no…drawbacks seemed to fade and it was just another way of conducting a visit. It felt very much like a face-to-face visit. [Site 1, psychiatrist]

Video has…better outcomes and a higher quality of care than phone, because you get more of the facial expression, or body movements and reactions…you just get more information. Even being able to see someone’s background and see where are they positioned, what does it look like at their home? Those types of things all provide valuable information. You don’t get that via the phone. [Site 1, psychologist]

Conversely, respondents at lower adoption sites more frequently questioned whether critical components of therapy are missing when seeing patients via video as opposed to in-person:

[TMH-V is] something new and exciting but at the same time, I’m also probably not as young and I do feel the value of human contact…[TMH-V] does connect people but it also isolates people. [Site 3, chief of psychiatry]

I think there’s something about being in [the same] room with a person…there’s a warmness to the connection that can happen… it’s a very subtle thing. [Site 3, psychologist]

Providers across sites expressed some concerns that it would be harder to manage high-risk situations via TMH-V versus in-person and that this may impact the quality of crisis management they would be able to offer, but most had not yet undergone this process with any of their patients. Providers also frequently noted instances of inappropriate behavior among their patients while on video (e.g., lying in bed, driving, doing chores); some were unsure if they should address these behaviors in the moment and questioned how this impacted the quality of the care they were able to provide as compared to in-person.

#### Compatibility

Respondents at Site 1 (higher adoption site) were more likely to discuss how providing care via TMH-V made sense for their patient population and fit smoothly within their clinical workflows:

I jumped into [TMH-V] with both feet because a lot of my visits are for Suboxone maintenance and [patients] have to come frequently and they’re scattered all over [the region] so –it was just such a nice fit for what I do. [Site 1, psychiatrist]

One provider at Site 1 noted that TMH-V had been written into their job description even prior to the pandemic. In contrast, respondents at the lower adoption sites were more likely to express beliefs that TMH-V, and particularly its scheduling and documentation requirements, were incompatible with preexisting systems:

It’s just a logistical nightmare…every person has three clinics: an in-person clinic, a telephone clinic, a [TMH-V] clinic… we come up with these crazy things where you only have one [TMH-V] visit per day and then you have to overbook, and so that messes up our numbers. [Site 4, mental health service chief]

#### Leadership engagement

At the two higher adoption sites, there was near unanimous endorsement of mental health leadership being strongly supportive of TMH-V. Leaders provided clear messaging that TMH-V should be prioritized during the pandemic, particularly over phone care, and also worked quickly to ensure that providers had access to necessary equipment.

We have new leadership in mental health…and she has done a phenomenal job and I think that’s made a big difference… they always promote [TMH-V] and don’t make it sound like it’s going to be a great big pain to do… [Site 2, facility telehealth coordinator]

At the lower adoption sites, respondents described a greater push for providers to return to in-person care during the pandemic; several providers took issue with this decision due to safety concerns and beliefs that they could provide high quality care via video.

We’re getting conflicting messages and changing messages pretty rapidly… it’s been confusing…there’s been concerns that our facility leadership is not making the distinction that Mental Health can deliver a service as equally well virtually as in person. So when [local] mandates come down that you need to do face-to-face [instead of TMH-V], there’s been kind of a disconnect there. [Site 3, psychiatrist]

This decision to prioritize in-person care seemed driven in part by leadership’s skepticism of TMH-V effectiveness research; the mental health service chief at Site 4 explained that “telehealth hasn’t been around long enough, and it’s maybe not the best quality literature.”

#### Champions

Respondents at the higher adoption sites identified specific champions of TMH-V; at Site 1, the mental health lead was a vocal and involved supporter of TMH-V who believed strongly in its value and described going door to door to help providers:

I personally was pretty excited about [TMH-V]…you need a champion…that is in the trenches, get them excited about it and then once somebody down the hall’s like, ‘Oh I heard, you know, [so-and-so] was doing this and it’s working,’ they’ll do it…I started walking down the hallways and sitting with people one-on-one and getting them up and running. [Site 1, mental health lead]

Site 2 noted that one of their telehealth technicians was extremely responsive and quick to help when problems emerged; the psychology lead at this site also described there being several psychologists and social workers “who keep up with everything [related to TMH-V], and when they get more information or find out new tricks of the trade they will share it with everybody.” Conversely, champions were not identified at the two lower adoption sites. Leadership at Site 4 noted that telehealth technicians had been moved into a different service line and were not as accessible to their providers; they noted that these technicians might have served as valuable resources and TMH-V champions if this shift had not occurred.

Maybe we haven’t used [the telehealth technicians] enough because I do think that…early adopters…people who really are cheerleaders might be more effective at communicating [about TMH-V] than me. [Site 4, mental health service chief]

## Discussion

The current study used the CFIR to guide analysis of barriers and facilitators to TMH-V uptake within VA during COVID-19. Five constructs had positive overall influences across sites: Relative advantage, Patient needs and resources, Relative priority, Knowledge and beliefs, and Self-efficacy. Complexity had a negative overall influence. Four constructs emerged as significant differentiators between high and low adoption sites: Quality, Compatibility, Leadership engagement, and Champions.

With regards to positive overall influences, respondents across all sites acknowledged the relative advantages of TMH-V including convenience for patients; removing the need to travel to appointments was cited as a particular strength that eliminated substantial access barriers. They also noted patients’ generally high satisfaction with this mode of care delivery, provided there were not significant difficulties connecting. Respondents also agreed that TMH-V was a relatively high organizational priority due to pandemic-related restrictions, which also encouraged uptake. In addition, as has been noted in prior work [19], providers’ attitudes towards TMH-V improved as they gained experience and self-efficacy navigating the technology. In contrast, complexity served as a negative overall influence across sites, such that providers and leadership described components of the TMH-V scheduling and troubleshooting processes as being unwieldy. There is still work to be done to optimize TMH-V platforms and make them as simple and user-friendly as possible for both patients and providers. There is a need for increased resources, in terms of ensuring that all patients can access video-enabled devices, adequate broadband connectivity, and technical support, including pre-visit test calls. This will be critical in attempting to close the well-documented digital divide, in which older and lower-income patients, as well as older providers, may have lower access to or comfort navigating TMH-V technologies [14, 26, 27].

The emergence of Quality, Leadership engagement, and Champions as key differentiators speaks to the importance of educating frontline staff and leadership at lower adoption sites about the well-established evidence base demonstrating that TMH-V is high-quality care [20, 28–31]. Stakeholders at higher adoption sites were more likely to view TMH-V as being largely equivalent to in-person care and in turn to champion its use; in this sense, they saw the shift towards telehealth during the pandemic as an opportunity to improve access to high-quality care via new modes of care delivery. Conversely, providers and leadership at lower adoption sites were more likely to be skeptical of the quality of TMH-V, viewing its use as more of an emergency, stopgap measure and encouraging a return to more in-person services as soon as their hospitals lifted pandemic-related restrictions. Continuing to disseminate information to leadership and providers about the effectiveness of TMH-V, as well as high reported levels of both patient and provider satisfaction [4–6, 8, 9, 11], will be critical in influencing attitudes regarding TMH-V quality, which may in turn increase rates of uptake.

Compatibility also emerged as a key differentiator; if TMH-V is not easily integrated into workflows, uptake will falter [15]. Higher adoption sites were notable for having more hands-on support from champions and telehealth technicians around complex processes including scheduling and troubleshooting, while lower adoption sites struggled to manage TMH-V logistics, frustrating providers and discouraging TMH-V use. Given that members of higher-level leadership may have control over resources being provided to support TMH-V care, it may be particularly important to target the abovementioned education efforts regarding the high quality of TMH-V towards these individuals; shifting beliefs regarding the positive value of TMH-V may in turn lead to improvements in the infrastructure needed to ensure a seamless and positive user experience for both patients and providers.

The current work is limited by its restriction to four VA sites. While a major strength of qualitative research is its ability to conduct rich, nuanced analyses, it also limits generalizability to other populations and healthcare systems. For instance, VA had a TMH-V infrastructure in place prior to COVID-19 as it was not subject to the same licensure and reimbursement restrictions of private healthcare systems that had largely disincentivized telehealth use pre-pandemic [3, 32–34]; future research should examine similar questions of TMH-V uptake across non-VA sites. In addition, the degree of variation in TMH-V use between high and low sites was somewhat restricted (range= 83.6–97.7%), given the overall increase in TMH-V use during the pandemic. Our work also does not assess patient perspectives, including their beliefs regarding the quality of TMH-V, the degree of choice they have in how they receive mental health care, and what type of technical support they feel is most helpful. Critical work has begun in this domain [9, 10] and additional rigorous research is needed.

Our interview guides were informed by CFIR domains and constructs. Although we feel that the CFIR encompasses many key components of implementation, it has been acknowledged that it may lack precision in certain areas (e.g., considerations of health equity), which may in turn have influenced the findings of our current work [35]. We note, however, that while we included questions specific to CFIR, our interview guide was semi-structured and offered opportunities for participants to respond to more open-ended questions regarding facilitators and barriers to TMH-V use. Finally, as is always the case within qualitative research, there is the potential for bias on the part of the analytic team. SLC and CJM are MH providers who both use TMH-V technology with their patients, which could serve as both a strength with regards to understanding contextual factors, but could also influence interpretation of results. JLS is not a MH provider and therefore helped to attenuate this potential for bias during analyses.

## Conclusions

The current study identified key facilitators and barriers to TMH-V uptake across higher and lower adoption VA sites. Findings point to the importance of education regarding the high quality of TMH-V care, the need for strong, hands-on support from leadership and champions, and the seamless integration of TMH-V into clinical workflows in order to ensure implementation success. Ideally, future work can draw from these findings to develop implementation strategies to increase TMH-V uptake at lower adoption sites, ensuring access for all those who stand to benefit from this innovative and high-quality mode of mental health care delivery.

## Supplementary Information

**Additional file 1.** Interview Guides for Providers, Leadership, and Telehealth Technicians/Coordinators.

**Additional file 2.** List of codes.

**Additional file 3.** Damschroder et al. criteria to assign ratings to constructs.

**Additional file 4.** SRQR checklist.

## Data Availability

The datasets generated and/or analyzed during the current study are not publicly available due to the potential of the privacy of the interviewees being compromised.

## Abbreviations

CFIR: Consolidated Framework for Implementation Research
COVID-19: Coronavirus disease 2019
MH: Mental health
PTSD: Posttraumatic stress disorder
SRQR: Standards for Reporting Qualitative Research
TMH-V: Telemental health via videoconferencing
VA: Department of Veterans Affairs
VSSC: Veterans Health Administration Support Service Center

## Footnotes

1 Note: High and low adoption sites were initially chosen in February 2020, and 4 interviews were conducted at Site 1 in March 2020; however, the onset of COVID-19 led to a subsequent four-month pause in interview recruitment given the high demands placed on MH staff in responding to the pandemic. The percentage of providers who completed a TMH-V visit was reassessed in June 2020 to confirm site selection; Site 1 remained the highest adoption site so the 4 interviews conducted at this site in March were retained. The two lower adoption sites changed from the initial February 2020 selection. Recruitment resumed in July 2020.

2 We considered including information regarding providers being high and low utilizers of TMH-V based on the VSSC data. We ultimately decided against this as providers at Site 1 had lower total TMH-V utilization as compared to providers at the remaining three sites, given that Site 1 providers were interviewed during the first few weeks of the pandemic (March 2020), while the remaining providers were interviewed in the summer and fall of 2020.
