# Supplementary material for: Factors influencing uptake of telemental health via videoconferencing at high and low adoption sites within the Department of Veterans Affairs during COVID-19: a qualitative study
Source: Implement Sci Commun. 2022 Jun 20;3:66. doi: 10.1186/s43058-022-00318-x (PMC9207848; doi:10.1186/s43058-022-00318-x)
Supplement: Supplementary file 1 — Additional file 1. Interview Guides for Providers, Leadership, and Telehealth Technicians/Coordinators. [file 43058_2022_318_MOESM1_ESM.docx]

Additional File 1. Interview Guides for Providers, Leadership, and Telehealth Technicians/Coordinators

General comment: These are intended to serve as guides for the semi-structured qualitative interviews conducted with providers, leadership, and telehealth technicians and coordinators.

1. **Draft Interview Guide for Providers**

**First, we’d like to learn about you and your role at this VAMC.**

- How long have you been at this medical center?
- What is your role in the facility?
- How long have you been in your current position?

***Now I’d like to ask some questions about VA Video Connect and virtual care.***

- How long have you been conducting VVC appointments?
- What prompted you to first start using VVC?
- Approximately what percentage of your appointments are currently delivered via VVC/telephone/in-person with masks?

**Intervention characteristics**

- Currently, how do you decide whether to see a patient via VVC, phone, or in-person?
  - [Probe for circumstances in which they may favor one modality over the other, e.g. certain patient populations, clinical scenarios]
  - **Probe for how you are ‘selling’ phone vs video**
- In general, how do you think the quality of care delivered via VVC compares to:
  - telephone care
  - in-person with masks
  - pre-COVID in-person appointments
- What have been your experiences with things like equipment set up, scheduling sessions, and conducting sessions with patients via VVC?
  - [Probe for technical problems]
- Have you conducted remote MH appointments from home?
  - If No, probe as to why not.
  - If Yes: what have been your experiences conducting MH appointments from home? [probe for challenges]
  - How would you feel about continuing to conduct virtual MH appointments from home for the long-term?

Outer setting

- Do your patients seem interested in using VVC? Do any prefer telephone appointments? Have any of your patients chosen to postpone treatment until it can be resumed in person?
  - Probe for differences between patients falling into these categories
- Thinking more broadly, how well do you feel VA has responded to the need to shift mental health care to virtual formats on a national or regional level?
- Are you aware of any national or regional guidance regarding mental health providers conducting VVC versus telephone versus in-person appointments?

If yes, has this influenced the type of care you have been providing over recent months?

Inner setting

- Can you share with me how your clinic’s use of VVC has evolved over time?

Probes:

- How would you describe your clinic/service’s willingness to try VVC?
- **Does VVC fit within your existing workflows and systems**?
- **How much has VVC use been prioritized within your clinic/service as compared to completing telephone appointments** or in-person appointments with masks? Are there any incentives for you to complete VVC appointments?
- How would you describe **leadership involvement** in VVC use?
- What type of resources have been available to support use of VVC appointments?
  - [Probe: access to a private room with a webcam/availability of VA-issued equipment for home use/help with scheduling/tech support]
- Have you had adequate access to information about VVC, such as trainings, handouts, contact info, and other necessary materials?
  - IF YES: Is there anything else you would like to have had information on?
  - IF NO: What else would you have liked?
- **Do you feel that your patients have adequate access to information about VVC,** such as educational materials, **test calls, tech support**?
- **Do you feel that you have had enough training in how to conduct therapy remotely**; e.g. developing therapeutic alliance, addressing therapy-interfering behaviors?
- **Disinhibition**
- **Wearing masks?**
- **No show rates?**

Process

- Are you aware of any staff members who you think are advocates for VVC use? If so, describe who they are and how they have been involved.

Characteristics of individuals

- How comfortable are you using new technology such as VVC? How similar is this to other technology you have used?
- How do you view VVC in terms of safety and security when providing care?

Overall facilitators/barriers

- What, if anything, has facilitated your use of VVC with your patients/ use of VVC overall within your clinic/service?
- What, if anything, has gotten in the way of you using VVC with your patients/ use of VVC overall within your clinic/service?
- Is there anything you would change about how VVC is being rolled out at your site?
- Is there anything you would change about the VVC platform itself?
- Are there alternative telehealth platforms that you prefer?
  - If yes, why?

Demographic differences

- Do you think there are differences in the types of patients being approached for/agreeing to VVC sessions as compared to the general patient population in your clinic/site? [E.g., differences in age, SES, or diagnosis.] If so, why?
- Do you think there are differences in the types of providers who prefer VVC versus telephone or in-person appointments in your clinic? [E.g. discipline, age] If so, why?
- **Moving forward?**

Wrap-up

- Is there anything else you’d like us to know about your opinions/experiences using VVC?
- Do you have any questions for me?
- Are there other people at your site that you recommend I speak to?
- Thank you very much for taking the time to participate. Your opinions will be very valuable as we try to understand how VVC is being used across mental health clinics within VA.

1. **Draft Interview Guide for Mental Health Leadership**

**First, we’d like to learn about you and your role at this VAMC.**

- How long have you been at this medical center?
- What is your role in the facility?
- How long have you been in your current position?

***Now I’d like to ask some questions about VA Video Connect and virtual care.***

- How familiar are you with VVC technology?
- To your knowledge, approximately when did providers at your facility start using VVC?
- Have you attended any trainings or used VVC in any capacity yourself? Have you seen what the program looks like?

**Intervention characteristics**

- How much in person/phone/video care
- In general, how do you think the quality of care delivered via VVC compares to:
  - telephone care
  - in-person with masks
  - pre-COVID in-person appointments
- How do you determine who gets what?
- What is your sense of how things have been going in terms of equipment set up, scheduling sessions, and conducting sessions with patients via VVC?

[probe for technical problems]

- Have providers at your site/clinic been conducting MH appointments from home?
  - If Yes: what is your sense of how this has been going?
  - How would you feel about providers continuing to conduct virtual MH appointments from home for the long-term?

**Outer setting**

- Do your patients at your site/clinic seem interested in using VVC? Do they prefer telephone appointments? Have patients chosen to postpone treatment until it can be resumed in person?
  - Probe for differences between patients falling into these categories
- On a broader level, how well do you feel VA has responded to the need to shift mental health care to virtual formats, nationally or regionally?
- Are you aware of any national or regional guidance regarding mental health providers conducting VVC versus telephone versus in-person appointments?

If yes, has this influenced the type of care your clinic/site has been providing over recent months?

**Inner setting**

- Can you share with me how your clinic’s use of VVC has evolved over time?

Probes:

- Given the many priorities and initiatives you must manage within your clinic/site, where do you feel VVC implementation falls in terms of importance and degree of effort allocated?
- How would you describe your clinic/service’s willingness to try VVC?
- Does VVC fit within your existing workflows and systems?
- Do you think your providers are interested in using VVC or see a need for it?
- How do you feel this initiative has been communicated to providers? Are there any incentives for providers to complete VVC appointments versus telephone or in-person appointments?
- How would you describe leadership involvement in in VVC use?
- What type of resources have been available to support use of VVC appointments?
  - [Probe: access to a private room with a webcam/availability of VA-issued equipment for home use/help with scheduling/tech support]
- Do stakeholders at your clinic/site have adequate access to information about VVC, such as trainings, handouts, contact info, and other necessary materials?
  - IF YES: Is there anything else you would like to have had information on?
  - IF NO: What else would you have liked?
- Do you feel that patients have adequate access to information about VVC, such as educational materials, test calls, tech support?
- **Disinhibition**
- **Wearing masks?**
- **No show rates?**

**Process**

- Has any planning taken place about ways to use VVC in your clinic/site?
  - If yes, how do you think the planning/implementation process has been going?
  - If no, what do you think are some reasons for this?
- Have any individuals been specifically designated as part of the VVC implementation effort, such as clinical champions or facilitators?
  - If yes, how do you think are they doing?
  - If no, explain why that is the case [e.g., not enough bandwidth to take on the

role, no one has been approached, etc]

**Characteristics of individuals**

- How comfortable are you using new technology such as VVC? How similar is this to other technology you have used?
- How do you view the safety and security of VVC in providing care?

**Overall facilitators/barriers**

- What, if anything, has facilitated the use of VVC within your clinic/service?
- What, if anything, has gotten in the way of VVC being used within your clinic/service?
- Is there anything you would change about how VVC is being rolled out at your site?
- Is there anything you would change about the VVC platform itself?
- Are there alternative telehealth platforms that you prefer?
  - If yes, why?

**Demographic differences**

- Do you think there are differences in the types of patients being approached for/agreeing to VVC sessions as compared to the general patient population in your clinic/site? [E.g., differences in age, SES, or diagnosis.] If so, why?
- Do you think there are differences in the types of providers completing VVC appointments in your clinic? [E.g. discipline, age] If so, why?

**Wrap-up**

- Is there anything else you’d like us to know about your opinions/experiences using VVC?
- Do you have any questions for me?
- Are there other people at your site that you recommend I speak to?
- Thank you very much for taking the time to participate. Your opinions will be very valuable as we try to understand how VVC is being used across mental health clinics within VA.

1. **Draft Interview Guide for Facility Telehealth Coordinators**

**First, we’d like to learn about you and your role at this VAMC.**

- How long have you been at this medical center?
- What is your role in the facility?
- How long have you been in your current position?

**VVC experience**

- How long have you been working with VVC? How about other forms of video telehealth?
- What services do you work with [e.g. mental health, primary care, telederm, teleretinal]? How much of your time is spent working with mental health?
- What tasks do you complete related to VVC use? [e.g. test calls, equipment installation, provider trainings, troubleshooting]

**Intervention characteristics**

- What have your experiences been with VVC in terms of equipment set up, scheduling sessions, providers conducting sessions? [probe for tech issues]
  - What are the most common VVC-related tasks that you spend time on?
- In general, how do you think the quality of care delivered via VVC compares to:
  - telephone care
  - in-person with masks
  - pre-COVID in-person appointments

**Outer setting**

- Have you interacted with patients related to your VVC work [e.g. test calls, patient outreach]?
  - If yes, do you get the sense that patients have an interest/need for VVC?
- Are you aware of any national or regional VA directives regarding mental health providers’ use of VVC versus telephone appointments or in-person with masks?
  - If yes, do you feel that this has had an influence on use of VVC in your facility?

**Inner setting**

- Can you share with me how your site’s use of VVC has evolved over time?

**Probes:**

- How would you describe the MH service/clinics willingness to try VVC?
- Do you feel that VVC fits within existing workflows and systems within mental health?
- Do you feel that VVC use has been prioritized within mental health at your site?
- How would you describe mental health leadership involvement in VVC use?
- What type of resources have been available to support use of VVC appointments?
  - [Probe: access to a private room with a webcam/availability of VA-issued equipment for home use/help with scheduling/tech support]
- Do you feel that stakeholders at your site have adequate access to information about VVC, such as trainings, handouts, contact info, and other necessary materials?
  - IF YES: Is there anything else you would like to have had information on?
  - IF NO: What else would you have liked?
- Do you feel that patients at your site have adequate access to information about VVC, such as educational materials, test calls, tech support?

**Process**

- Are you aware of any staff members who you think are advocates for VVC use? If so, describe who they are and how they have been involved.

**Characteristics of individuals**

- How comfortable are you using new technology such as VVC? How similar is this to other technology you have used?
- How do you view the safety and security of VVC in providing care?

**Overall facilitators/barriers**

- What, if anything, has facilitated VVC use within the mental health service at your site?
- What, if anything, has gotten in the way of VVC use within the mental health service at your site?
- Is there anything you would change about how VVC is being rolled out within mental health at your site?
- Is there anything you would change about the VVC platform itself?
- Do you prefer an alternative telehealth platform?
  - If yes, why?

**Demographic differences**

- Do you think there are differences in the types of patients being approached for/agreeing to VVC sessions as compared to the general mental health patient population at your site? [E.g., differences in age, SES, or diagnosis.] If so, why?
- Do you think there are differences in the types of mental health providers completing VVC appointments at your site? [E.g. discipline, age] If so, why?

**Wrap-up**

- Is there anything else you’d like us to know about your opinions/experiences using VVC?
- Do you have any questions for me?
- Are there other people at your site that you recommend I speak to?
- Thank you very much for taking the time to participate. Your opinions will be very valuable as we try to understand how VVC is being used across VA mental health services.
