# Supplementary material for: Factors influencing uptake of telemental health via videoconferencing at high and low adoption sites within the Department of Veterans Affairs during COVID-19: a qualitative study
Source: Implement Sci Commun. 2022 Jun 20;3:66. doi: 10.1186/s43058-022-00318-x (PMC9207848; doi:10.1186/s43058-022-00318-x)
Supplement: Supplementary file 2 — Additional file 2. List of codes. [file 43058_2022_318_MOESM2_ESM.docx]

Additional File 2. List of codes

CFIR code definitions are taken from cfirguide.org; any project-specific modifications to definitions are italicized.

| **CFIR CODES** | | |
| --- | --- | --- |
| **Characteristics of individuals** | Knowledge and beliefs | Individuals’ attitudes toward and value placed on the intervention as well as familiarity with facts, truths, and principles related to the intervention |
|  | Self-efficacy | Individual belief in their own capabilities to execute courses of action to achieve implementation goals. *(e.g., level of tech proficiency and comfort)* |
| **Intervention characteristics** | Complexity | Perceived difficulty of the intervention (e.g. intricacy and number of steps required to implement- *includes tech glitches/complications)* |
|  | Quality | Stakeholders’ perceptions of the quality and validity of evidence supporting the belief that the intervention will have desired outcomes. (*including how quality of video telehealth compares to in-person or phone care; ways in which remote care can introduce more distraction/casualness on part of patient and provider, safety/security)* |
|  | Relative advantage | Stakeholders’ perception of the advantage of implementing the intervention versus an alternative solution *(e.g., phone or in-person care)* |
| **Inner setting** | Access to knowledge and information | Ease of access to digestible information and knowledge about the intervention and how to incorporate it into work tasks. |
|  | Available resources | The level of resources dedicated for implementation and on-going operations, including money, training, education, physical space, and time. |
|  | Networks and communication | The nature and quality of webs of social networks and the nature and quality of formal and informal communications within an organization. |
|  | Compatibility | The degree of tangible fit between meaning and values attached to the intervention by involved individuals, how those align with individuals’ own norms, values, and perceived risks and needs, and how the intervention fits with existing workflows and systems. |
|  | Culture | Norms, values, and basic assumptions of a given organization. |
|  | Goals and feedback | The degree to which goals are clearly communicated, acted upon, and fed back to staff, and alignment of that feedback with goals. |
|  | Implementation climate | The absorptive capacity for change, shared receptivity of involved individuals to an intervention, and the extent to which use of that intervention will be rewarded, supported, and expected within their organization. |
|  | Leadership engagement | Commitment, involvement, and accountability of leaders and managers with the implementation. |
|  | Incentives and rewards | Extrinsic incentives such as goal-sharing awards, performance reviews, promotions, and raises in salary, and less tangible incentives such as increased stature or respect. (*includes potential differences in workload credit for phone versus video)* |
|  | Relative priority | Individuals’ shared perception of the importance of the implementation within the organization. |
| **Outer setting** | External policies & incentives | Policy and regulations (governmental or other central entity), external mandates, recommendations and guidelines, pay-for-performance, collaboratives, and public or benchmark reporting. |
|  | Patient needs and resources | The extent to which patient needs, as well as barriers and facilitators to meet those needs, are accurately known and prioritized by the organization. |
| **Process** | Champions, opinion leaders | Champions: Individuals who dedicate themselves to supporting, marketing, and ‘driving through' implementation. Opinion leaders: those w/ formal or informal influence on the attitudes and beliefs of their colleagues re: implementing the intervention. |
|  | Planning, engaging, executing | Planning: The degree to which a scheme or method of behavior and tasks for implementing an intervention are developed in advance, and the quality of those schemes or methods. Engaging: Attracting and involving appropriate individuals in the implementation and use of the intervention through a combined strategy of social marketing, education, role modeling, training, and other similar activities. Executing: Carrying out or accomplishing the implementation according to plan. |
| **OTHER CODES** | | |
|  | *Barriers* | *Any significant barriers to implementation (e.g., tech glitches)* |
|  | *Facilitators* | *Any significant facilitators to implementation (e.g., helpful telehealth staff)* |
|  | *COVID as catalyst* | *Concept that telehealth implementation was catalyzed by COVID in-person restrictions* |
|  | *Golden nuggets* | *Particularly strong/representative quotes* |
|  | *Respondent background* | *Position title, length of time worked at organization* |
|  | *Respondent telehealth experience* | *Extent of telehealth experience, includes percentage of care delivered via telehealth, working from home, use of various platforms.* |
|  | *Modality choice* | *How the provider chooses whether to provide care via phone, video, or in-person/how they present it to the patient* |
|  | *Future plans* | *How the provider would want to incorporate telehealth/work from home into long-term work* |
|  | *Provider differences* | *Differences in telehealth use based on provider characteristics (e.g., age, discipline.)* |
