# Supplementary material for: Factors influencing uptake of telemental health via videoconferencing at high and low adoption sites within the Department of Veterans Affairs during COVID-19: a qualitative study
Source: Implement Sci Commun. 2022 Jun 20;3:66. doi: 10.1186/s43058-022-00318-x (PMC9207848; doi:10.1186/s43058-022-00318-x)
Supplement: Supplementary file 3 — Additional file 3. Damschroder et al. criteria to assign ratings to constructs. [file 43058_2022_318_MOESM3_ESM.docx]

**Additional File 3. Damschroder et al. criteria to assign ratings to constructs**

From: Damschroder, L. J., & Lowery, J. C. (2013). Evaluation of a large-scale weight management program using the consolidated framework for implementation research (CFIR). *Implementation Science*, *8*(1), 1-17.

| −2 | The construct is a negative influence in the organization, an impeding influence in work processes, and/or an impeding influence in implementation efforts. The majority of interviewees (at least two) describe explicit examples of how the key or all aspects (or the absence) of a construct manifests itself in a negative way. |
| --- | --- |
| −1 | The construct is a negative influence in the organization, an impeding influence in work processes, and/or an impeding influence in implementation efforts. Interviewees make general statements about the construct manifesting in a negative way but without concrete examples: |
|  | • The construct is mentioned only in passing or at a high level without examples or evidence of actual, concrete descriptions of how that construct manifests; |
|  | • There is a mixed effect of different aspects of the construct but with a general overall negative effect; |
|  | • There is sufficient information to make an indirect inference about the generally negative influence; and/or |
|  | • Judged as weakly negative by the absence of the construct. |
| 0 | A construct has neutral influence if: |
|  | • It appears to have neutral effect (purely descriptive) or is only mentioned generically without valence; |
|  | • There is no evidence of positive or negative influence; |
|  | • Credible or reliable interviewees contradict each other |
|  | • There are positive and negative influences at different levels in the organization that balance each other out; and/or different aspects of the construct have positive influence while others have negative influence and overall, the effect is neutral. |
| +1 | The construct is a positive influence in the organization, a facilitating influence in work processes, and/or a facilitating influence in implementation efforts. Interviewees make general statements about the construct manifesting in a positive way but without concrete examples: |
|  | • The construct is mentioned only in passing or at a high level without examples or evidence of actual, concrete descriptions of how that construct manifests; |
|  | • There is a mixed effect of different aspects of the construct but with a general overall positive effect; and/or |
|  | • There is sufficient information to make an indirect inference about the generally positive influence. |
| +2 | The construct is a positive influence in the organization, a facilitating influence in work processes, and/or a facilitating influence in implementation efforts. The majority of interviewees (at least two) describe explicit examples of how the key or all aspects of a construct manifests itself in a positive way. |
| Missing | Interviewee(s) were not asked about the presence or influence of the construct; or if asked about a construct, their responses did not correspond to the intended construct and were instead coded to another construct. Interviewee(s) lack of knowledge about a construct does not necessarily indicate missing data and may instead indicate the absence of the construct. |
